# Trends and patterns of injuries among children under five in Mongolia: A retrospective analysis of national injury surveillance data between 2018 and 2022

**DOI:** 10.1111/tmi.14117

**Published:** 2025-05-08

**Authors:** Tsetsegee Sambuu, Emma M. Rath, Feiyu Hu, Tumen‐Ulzii Badarch, Yui Yumiya, Tatsuhiko Kubo, Oyunsuren Enebish, Odgerel Chimed‐Ochir

**Affiliations:** ^1^ Department of Public Health and Health Policy, Graduate School of Biomedical and Health Sciences Hiroshima University Hiroshima Japan; ^2^ Giannoulatou Laboratory Victor Chang Cardiac Research Institute Sydney New South Wales Australia; ^3^ College of Sustainability and Tourism Ritsumeikan Asia Pacific University Beppu Oita Japan; ^4^ Department of Statistics and Surveillance National Trauma and Orthopedic Research Center Ulaanbaatar Mongolia; ^5^ Department of Public Health Policy Ministry of Health Ulaanbaatar Mongolia

**Keywords:** epidemiology, injuries, Mongolia, preschool child

## Abstract

**Objective:**

This retrospective study aimed to analyse the pattern and trend of fatal and non‐fatal injuries among children under five in Mongolia from 2018 to 2022, using hospital‐based national injury surveillance data to inform targeted public health interventions.

**Methods:**

Data from 101,731 injury cases were analysed from the National Trauma and Orthopaedic Research Center's surveillance system. Injury incidence and mortality rates were calculated based on demographic characteristics and geographic distributions.

**Results:**

From 2018 to 2022, 101,731 children under five sustained injuries in Mongolia, with most occurring at home (78.9%) and in Ulaanbaatar (87.6%). Boys accounted for 55.7%. Ulaanbaatar had the highest non‐fatal injury incidence rate (1003 per 100,000), primarily from falls (45.8%), burns (16.5%) and mechanical forces. Non‐fatal injuries increased annually in Ulaanbaatar, especially among boys (6.6%) and girls (9.9%). Children aged 1–4 years and boys were at higher risk, with injuries more frequent in summer and at home.

Fatal injuries (*n* = 715) were mostly due to suffocation (34.7%), traffic (20.7%) and drowning (13.7%). Ulaanbaatar saw a significant decrease in fatality rates (14.9% annually for boys), while traffic‐related deaths rose in girls in provinces (18.6%). Mortality rates were higher among infants aged 0–11 months.

**Conclusion:**

There is an increase in non‐fatal injuries among children under 5 years of age in Mongolia, particularly in Ulaanbaatar, despite a declining trend in fatal injury rates. Injury rates also vary significantly across rural provinces, highlighting the need for geographically tailored policy interventions.

## INTRODUCTION

Globally, in 2021, nearly 39 million children under five were injured, and 200,000 lost their lives due to injuries, accounting for 4% of all under‐five mortality worldwide [1].

In Mongolia, the primary causes of under‐five mortality have shifted from communicable to non‐communicable diseases, with injuries now being the predominant cause. For infants, injuries rank as the third leading cause of death [2]. Alarmingly, the injury‐related mortality rate in Mongolia is 5.5 times higher than the global average and twice as high as that of other low‐middle‐income countries [1]. As of 2023, injuries accounted for 20.8% of all under‐five deaths in provincial areas and 7.8% in Ulaanbaatar [3]. Mongolia's transition from a nomadic to an urbanised society has driven over 80% of the population to urban areas, particularly Ulaanbaatar [2, 3,4]. This migration has resulted in densely populated informal settlements, presenting unique socioeconomic challenges that increase the risk of childhood injuries [5, 6, 7, 8]. Although extensive research on childhood injuries has been conducted in developed nations, data on injury patterns among children under five in Mongolia remain scarce.

Existing studies in Mongolia have typically focused on broader age groups or specific injury causes. For instance, research by Altangerel et al. studied potential years of life lost due to injuries among the working‐age population [9], while Pynn et al. highlighted high rates of childhood burns in Ulaanbaatar's *ger* areas [10]. Other studies examined and reported burns among children aged 15 years and younger in 2015–2016 [11] and children 0–18 years old in 2022 [2]. While valuable, these studies lack a comprehensive analysis of recent trends, causes and demographic or geographic patterns of injuries specific to children under five.

The first 5 years of life are critical for mental, physical and socio‐emotional development, forming the foundation for future health, education and economic productivity. Adverse events during this period can have profound, long‐term effects on a child's development and well‐being [12]. Under‐five mortality serves as a key indicator of population health, reflecting the quality of maternal, newborn and child health programmes, and is closely tied to the Sustainable Development Goals [13].

Therefore, we aim to assess and analyse the incidence, causes and trends of fatal and non‐fatal injuries among children under five in Mongolia over the past 5 years. The study findings will inform policymakers, healthcare providers and community leaders in developing targeted, culturally appropriate interventions to create safer environments for Mongolia's youngest and most vulnerable citizens.

## METHODS

### Study setting

Mongolia has a population of approximately 3.5 million, nearly half of whom (around 1.7 million) reside in the capital city, Ulaanbaatar [15]. Of this urban population, more than 10% are children under 5 years old [14]. Administratively, Mongolia is divided into the capital city, Ulaanbaatar and 21 provinces (aimags), which are grouped into five regions.

Ulaanbaatar itself is subdivided into districts. In Ulaanbaatar, family clinics and district health centres primarily provide outpatient care for injuries. The National Trauma and Orthopaedic Research Centre (NTORC) is a tertiary teaching referral hospital that traditionally provides outpatient, inpatient and emergency trauma care at the national level. However, recent decentralisation efforts have expanded the capacity of district health centres and some other referral‐level hospitals to offer inpatient care as well. Additionally, some private hospitals provide both outpatient and inpatient care for injury‐related cases. Emergency cases are transported either by ambulance services or private vehicles, while non‐emergency cases are typically brought to hospitals by private transport.

At the provincial level, each province is divided into *soums*, the smallest administrative units. Each soum has a soum health centre, and each province has a provincial hospital. These facilities generally provide outpatient injury treatment. Soum health centres are classified into three levels A, B and C based on population size, distance from the provincial centre and travel time. A‐ and B‐level centres, typically located at soum junctions or in high‐risk areas such as border zones, major transport routes and regions with industrial or tourism activity, are equipped to provide care for injury cases [3]. Provincial hospitals also deliver emergency and inpatient trauma care. In addition, Mongolia maintains five Regional Diagnostic and Treatment Centres located in the western, central, northern, southern and eastern regions. These facilities serve as referral centres for diagnosing and managing emergency and injury‐related conditions [16, 17]. When provincial and regional facilities are unable to manage complex cases, patients are referred to the NTORC in Ulaanbaatar. However, in many instances, particularly in Ulaanbaatar, patients bypass lower‐level facilities and present directly to NTORC, the National Centre for Maternal and Child Health (for children under 18 years of age), or the National Emergency Centre of Toxicology (in case of some type of poisonings), often without a formal referral.

Soum populations range from fewer than 2000 to around 10,000 [18]. On average, the distance between a provincial hospital and a soum health centre is 101 kilometres [19]. However, many soums are over 300 km from provincial centres and connected primarily by unpaved roads, making patient transport challenging [20]. Emergency referrals are transported using both local ambulance services and long‐distance medical transport, depending on the need [21].

Trauma care is covered by the National Health Insurance System and offered free of charge nationwide [22].

### Data source

Since 2017, Mongolia's NTORC began electronically registering injuries. The dataset for 2017 was significantly smaller than for subsequent years, and the sharp increase in data volume from 2018 suggests incomplete reporting in 2017. Therefore, this study excludes 2017 data and focuses on injury surveillance data from January 2018 to December 2022.

The majority of the data (86.9%) originated from specialised hospitals such as NTORC, the National Centre for Maternal and Child Health, and the National Emergency Centre of Toxicology, followed by family clinics and soum hospitals (5.1%), provincial hospitals (4.6%), regional hospitals (2.5%), district hospitals (0.7%) and others, including private hospitals (0.2%). The dataset includes demographics, causes of injury, diagnostic codes, place of occurrence, medical service utilisation, referrals and mortality. Injury causes and diagnoses follow International Statistical Classification of Diseases and Related Health Problems (ICD‐10) Geographic Information System (codes: V01‐V99 (transport accidents), W00‐X59 (other external accidental causes), X85‐Y09 (assault) and Y10‐Y34 (undetermined intent).

Population data on children under five by age, sex and location were sourced from the National Statistics Office of Mongolia [23]. Live birth data for 2018–2022 were obtained from Health Indicators reports compiled by the Health Development Center of Mongolia [24, 25, 26, 27, 28].

### Ethical compliance

Data obtained were fully anonymised. Ethical approval to publish the findings was obtained from the Ethics Committee of the Graduate School of Biomedical and Health Sciences, Hiroshima University, Japan (Approval No. E2024‐0164).

### Statistical analysis

A retrospective descriptive and inferential analysis was conducted to assess the burden of injuries among children under five.

The incidence rate (per 10,000 children) and mortality rate (per 1000 live births) were calculated by year, month, demographic attributes, geographic distribution and injury location. Percentages of specific causes of non‐fatal and fatal injuries were analysed for Ulaanbaatar and provinces, focusing on the five leading causes among 17 non‐fatal and 16 fatal categories based on year, month, demographics, geography and injury location.

Joinpoint regression analysis (version 5.0.2) was used to determine trends in non‐fatal and fatal injury rates from 2018 to 2022 [29]. The regression model employed the logarithmic equation: log(*Ry*) = *b*0 + *b*1*y*. The average percentage change from year *y* to year *y* + 1 was calculated as follows:

$$\left\lfloor \frac{R_{y+1}-R_{y}}{R_{y}}\right\rfloor \times 100=\frac{\left\{e^{b_{0}+b_{1}\left(y+1\right)-}e^{b_{0}+b_{1}\left(y\right)}\right\}}{e^{b_{0}+b_{1}\left(y\right)}}\;\times \;100=\left(e^{b_{1}}-1\right)\times 100.$$

The average annual percentage change (AAPC) from 2018 to 2022 was calculated using a weighted average of the slope coefficients of the underlying joinpoint regression line with weights equal to the length of each segment over the intervals. If we denote $b_{i}$ as the slope coefficients for each segment in the desired range of years, and $w_{i}$ as the length of each segment in the range of years, then AAPC would be

$$\left\{\mathrm{Exp}\left(\frac{\sum w_{i}b_{i}}{\sum w_{i}}\right)-1\right\}\times 100.$$

Statistical significance was tested using a Monte Carlo permutation method [30].

Geographic Information System (GIS) analysis was employed to calculate and visualise provincial injury rates (per 10,000 children) using a choropleth map with colour‐coded intervals.

The relationship between the investigated factors and non‐fatal injuries was assessed using the incidence rate ratios (IRR) using a negative binomial model. The negative binomial model was chosen because of the presence of overdispersion in the data, as indicated by a likelihood ratio (LR) test with *α* = 0. For fatal injuries, a Poisson regression model was applied when overdispersion was not detected.

## RESULTS

### Total injuries

From 2018 to 2022, 101,731 children under 5 years old sustained injuries in Mongolia (Table 1). Most injuries (78.9%) occurred at home, with 87.6% of cases in Ulaanbaatar. Boys accounted for 55.7% of all injuries.

**TABLE 1:** Summary of non‐fatal and fatal injuries among children under five in Mongolia between 2018 and 2022.

	Non‐fatal injury		Fatal injury		Total injuries	
	Number of incidence	Incidence rate (per 10,000 U5 children)	Number of deaths	Mortality rate (per 1000 live births)	*N*	%
Year						
2018	18,021	460.7	177	2.28	18,198	17.9%
2019	19,584	507.8	154	1.97	19,738	19.4%
2020	20,039	525.1	150	1.97	20,189	19.8%
2021	20,133	536.2	110	1.54	20,243	19.9%
2022	23,239	634.2	124	1.89	23,363	23.0%
Month						
January	6698	35.2	32	0.09	6730	6.6%
February	6919	36.4	51	0.14	6970	6.9%
March	7883	41.5	42	0.11	7925	7.8%
April	8362	44.0	68	0.18	8430	8.3%
May	9670	50.9	63	0.17	9733	9.6%
June	10,140	53.4	85	0.23	10,225	10.1%
July	9875	52.0	83	0.22	9958	9.8%
August	9926	52.2	73	0.20	9999	9.8%
September	8840	46.5	56	0.15	8896	8.7%
October	8273	43.5	58	0.16	8331	8.2%
November	7161	37.7	51	0.14	7212	7.1%
December	7269	38.3	53	0.14	7322	7.2%
Gender						
Male	56,259	577.0	413	2.18	56,672	55.7%
Female	44,757	483.7	302	1.68	45,059	44.3%
Age						
0–11 months	12,363	334.0	269	0.73	12,632	12.4%
1–4 years	88,653	579.4	446	1.21	89,099	87.6%
Geographic area						
Ulaanbaatar	89,175	1002.8	169	0.87	89,344	87.8%
Provinces	11,841	117.1	546	3.10	12,387	12.2%
Location						
Home	79,878	420.3	390	1.06	80,268	78.9%
Outside	21,138	111.2	325	0.88	21,463	21.1%
**Total**	**1,01,016**	**531.6**	**715**	**1.94**	**1,01,731**	**100.0%**

*Note*: Rates are presented with one decimal place for non‐fatal injury rate and two decimal places for fatal injury rate to ensure precision. In the text, rates are rounded according to their magnitude for readability. Bold formatting has been used to distinguish total values.

Abbreviation: U5 children, under five children.

**FIGURE 1:**
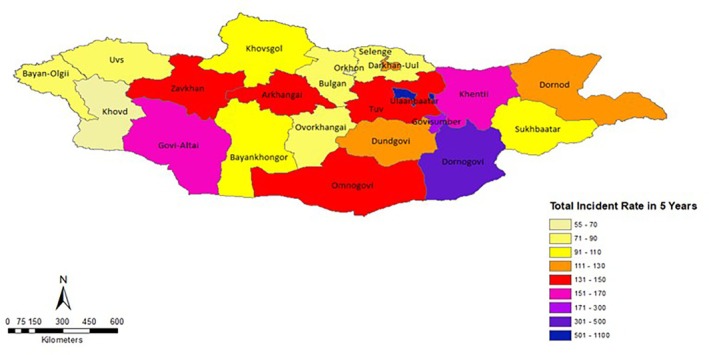
Map of injury rates in Mongolia: provincial and Ulaanbaatar city analysis.

Ulaanbaatar had the highest incidence rate (1004 per 10,000 person‐years), followed by Dornogovi (461), Govisumber (293) and Khentii (166). Provinces such as Khovd, Orkhon and Bayan‐Olgii reported lower rates, ranging from 57 to 72 per 10,000 person‐years (Figure 1).

### General characteristics and trends of non‐fatal injuries

A total of 101,016 non‐fatal injuries occurred, with an incidence rate of 532 per 100,000 under‐five children (Table 1). Rates were higher in Ulaanbaatar (1003 per 100,000) than in provinces (117 per 100,000). Injuries at home (420 per 100,000) were more frequent than outside the home (111 per 100,000). Falls were the leading cause (45.8%), followed by burns (16.5%), injuries from inanimate mechanical forces (13.6%), animate mechanical forces (12%) and traffic‐related injuries (3.7%), collectively accounting for 91.5% of all non‐fatal injuries (Table S1).

Falls often resulted from slipping or tripping (46.8%), while bed falls (23.0%) were equally distributed between Ulaanbaatar and provinces. Among injuries due to exposure to fire or hot objects, burns were primarily caused by hot drinks or food, accounting for 42.9% of cases in Ulaanbaatar and 59.0% in provinces. Animate mechanical injuries mainly involved accidental hits (86.2%) in Ulaanbaatar, whereas dog bites were more common (50.0%) in provinces. Injuries resulting from inanimate mechanical forces primarily resulted from being struck against or by objects, contributing to 49.7% of such injuries in Ulaanbaatar. Traffic‐related injuries were predominantly sustained by car occupants in both regions (Table S2).

Non‐fatal injuries were more common among boys and children aged 1–4 years, with an increased rate during summer and at home (Table 2).

**TABLE 2:** Five leading causes of non‐fatal injuries during 2018–2022 in Mongolia.

	Falls			Fire or hot object or substance			Struck by or against inanimate mechanical force			Exposure to animate mechanical forces			Traffic injury			Leading five causes		
	*N* ^a^	%^b^	IR^c^	*N* ^a^	%^b^	IR^c^	*N* ^a^	%^b^	IR^c^	*N* ^a^	%^b^	IR^c^	*N* ^a^	%^b^	IR^c^	*N* ^a^	%^b^	IR^c^
Gender																		
Male	26,011	46.2%	266.8	9489	16.9%	97.3	8220	14.6%	84.3	5834	10.4%	59.8	2097	3.7%	21.5	51,651	91.8%	529.7
Female	20,211	45.2%	218.4	7211	16.1%	77.9	5479	12.2%	59.2	6305	14.1%	68.1	1617	3.6%	17.5	40,823	91.2%	441.2
Age																		
0–11 months	5493	44.4%	148.4	3419	27.7%	92.4	841	6.8%	22.7	1093	8.8%	29.5	544	4.4%	14.7	11,390	92.1%	307.7
1–4 years	40,729	45.9%	266.2	13,281	15.0%	86.8	12,858	14.5%	84.0	11,046	12.5%	72.2	3170	3.6%	20.7	81,084	91.5%	529.9
Geographic area																		
Ulaanbaatar	41,721	46.8%	469.1	12,636	14.2%	142.1	12,241	13.7%	137.6	11,583	13.0%	130.2	2956	3.3%	33.2	81,137	91.0%	912.4
Provinces	4501	38.0%	44.5	4064	34.3%	40.2	1458	12.3%	14.4	556	4.7%	5.5	758	6.4%	7.5	11,337	95.7%	112.1
Location																		
Home	37,106	46.5%	195.3	15,522	19.4%	81.7	10,885	13.6%	57.3	9411	11.8%	49.5	140	0.2%	0.7	73,064	91.5%	384.5
Outside	9116	43.1%	48.0	1178	5.6%	6.2	2814	13.3%	14.8	2728	12.9%	14.4	3574	16.9%	18.8	19,410	91.8%	102.1
Season																		
Spring	11,749	45.3%	61.8	4344	16.8%	22.9	3674	14.2%	19.3	3097	12.0%	16.3	812	3.1%	4.3	23,676	91.4%	124.6
Summer	14,017	46.8%	73.8	4026	13.4%	21.2	4228	14.1%	22.2	3775	12.6%	19.9	1578	5.3%	8.3	27,624	92.3%	145.4
Autumn	10,983	45.2%	57.8	4153	17.1%	21.9	3183	13.1%	16.7	3002	12.4%	15.8	813	3.3%	4.3	22,134	91.2%	116.5
Winter	9473	45.4%	49.8	4177	20.0%	22.0	2614	12.5%	13.8	2265	10.8%	11.9	511	2.4%	2.7	19,040	91.2%	100.2
Total	46,222	45.8%	243.2	16,700	16.5%	87.9	13,699	13.6%	72.1	12,139	12.0%	63.9	3714	3.7%	19.5	92,474	91.5%	486.6

*Note*: ‘Undetermined intent’ was reported as the fifth leading cause of non‐fatal injuries; therefore, traffic injuries were included in its place to focus on specific injury categories. Spring—March, April, May; Summer—June, July, August; Autumn—September, October, November; Winter—December, January, February.

^a^ Number of incidents.

^b^ Percentage of injuries among all‐cause non‐fatal injuries.

^c^ Incidence rate (IR; per 10,000 under five children).

**FIGURE 2:**
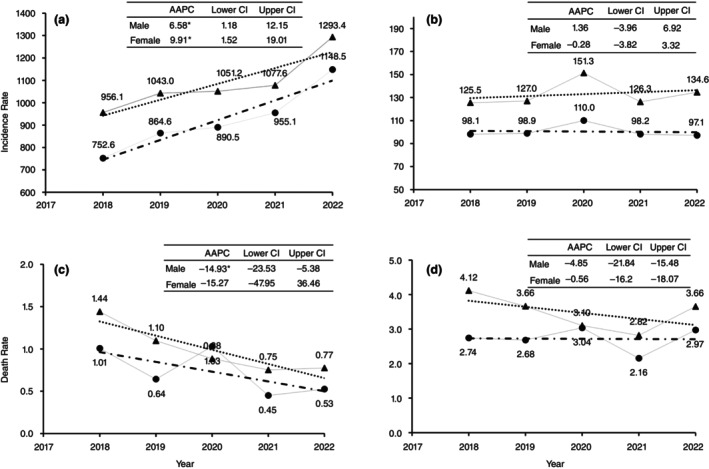
Trend of incidence rate and average annual percentage changes of non‐fatal and fatal injury during 2018–2022 in Mongolia. (a) Non‐fatal injury in Ulaanbaatar; (b) non‐fatal injury in provinces; (c) fatal injury in Ulaanbaatar; (d) fatal injury in provinces. *Significant Average Annual Percentage Changes of injuries between 2018 and 2022 (AAPC). Lower CI, lower confidence interval; Upper CI, upper confidence interval.

The non‐fatal injury rate significantly increased annually in Ulaanbaatar by 6.6% (95% confidence interval [CI] 1.2%–12.2%) for boys and 9.9% (95% CI 1.5%–19.0%) for girls but showed no significant change in provinces (Figure 2a,b). Among injury causes, inanimate mechanical forces increased significantly by 10.8% annually in girls (Figure S1C2), while exposure to animate mechanical forces rose by 30.8% in boys and 39.7% in girls in Ulaanbaatar (Figure S1D1).

### General characteristics and trends of fatal injuries

Between 2018 and 2022, 715 children under five died due to injuries, with a mortality rate of 2 per 1000 live births (Table 1). The fatal injuries accounted for less than 1% (0.7%) of all reported injuries (101,731). Mortality rates were higher in provinces (3.1) than in Ulaanbaatar (1 per 1000 live births).

Suffocation was the leading cause (34.7%), followed by traffic‐related injuries (20.7%), drowning (13.7%), burns (9.2%) and falls/poisoning (5.9% each), collectively contributing to 90.1% of deaths (Tables S1 and 3).

**TABLE 3:** Five leading causes of fatal injuries during 2018–2022 in Mongolia.

	Suffocation			Traffic injury			Drawning			Fire or hot object or substance			Falls			Poisoning			Leading five causes		
	*N* ^a^	%^b^	DR^c^	*N* ^a^	%^b^	DR^c^	*N* ^a^	%^b^	DR^c^	*N* ^a^	%^b^	DR^c^	*N* ^a^	%^b^	DR^c^	*N* ^a^	%^b^	DR^c^	*N* ^a^	%^b^	DR^c^
Gender																					
Male	143	34.6%	0.76	72	17.4%	0.38	63	15.3%	0.33	43	10.4%	0.23	30	7.3%	0.16	23	5.6%	0.12	374	90.6%	1.98
Female	105	34.8%	0.58	76	25.2%	0.42	35	11.6%	0.19	23	7.6%	0.13	12	4.0%	0.07	19	6.3%	0.11	270	89.4%	1.50
Age																					
0–11 months	198	73.6%	0.54	23	8.6%	0.06	5	1.9%	0.01	5	1.9%	0.01	10	3.7%	0.03	7	2.6%	0.02	248	92.2%	0.67
1–4 years	50	11.2%	0.14	125	28.0%	0.34	93	20.9%	0.25	61	13.7%	0.17	32	7.2%	0.09	35	7.8%	0.09	396	88.8%	1.07
Residence																					
Urban	54	32.0%	0.28	26	15.4%	0.13	8	4.7%	0.04	16	9.5%	0.08	18	10.7%	0.09	31	18.3%	0.16	153	90.5%	0.79
Rural	194	35.5%	1.10	122	22.3%	0.69	90	16.5%	0.51	50	9.2%	0.28	24	4.4%	0.14	11	2.0%	0.06	491	89.9%	2.79
Location																					
Home	204	52.3%	0.55	24	6.2%	0.06	18	4.6%	0.05	60	15.4%	0.16	22	5.6%	0.06	27	6.9%	0.07	355	91.0%	0.96
Outside	44	13.5%	0.12	124	38.2%	0.34	80	24.6%	0.22	6	1.8%	0.02	20	6.2%	0.05	15	4.6%	0.04	289	88.9%	0.78
Season																					
Spring	61	35.3%	0.17	36	20.8%	0.10	28	16.2%	0.08	13	7.5%	0.04	13	7.5%	0.04	9	5.2%	0.02	160	92.5%	0.43
Summer	56	23.2%	0.15	57	23.7%	0.15	56	23.2%	0.15	22	9.1%	0.06	16	6.6%	0.04	8	3.3%	0.02	215	89.2%	0.58
Autumn	66	40.0%	0.18	37	22.4%	0.10	12	7.3%	0.03	14	8.5%	0.04	8	4.8%	0.02	14	8.5%	0.04	151	91.5%	0.41
Winter	65	47.8%	0.18	18	13.2%	0.05	2	1.5%	0.01	17	12.5%	0.05	5	3.7%	0.01	11	8.1%	0.03	118	86.8%	0.32
Total	248	34.7%	0.67	148	20.7%	0.40	98	13.7%	0.27	66	9.2%	0.18	42	5.9%	0.11	42	5.9%	0.11	644	90.1%	1.74

^a^ Number of injury deaths.

^b^ Percentage of injury among all‐cause fatal injuries.

^c^ Death rate (DR; per 1000 live births).

Suffocation deaths were associated with incidents in bed, occurring at similar rates in Ulaanbaatar (50.0%) and provinces (52.6%). Traffic‐related injuries predominantly involved pedestrian injuries (84.5%), with similar rates in Ulaanbaatar and provinces. Drowning primarily occurred due to falls into natural water, with a higher proportion in Ulaanbaatar (75.0%) than in provinces (34.4%). Among injuries due to fire or hot objects, burns with hot drinks or foods were common in Ulaanbaatar (68.8%), while exposure to direct fire was higher in provinces (36.0%). Falls from structures (72.2%) in Ulaanbaatar were the main cause of death from falls (Table S3).

The overall fatal injury incidence rate declined significantly in Ulaanbaatar, with an average annual decrease of 14.9% (95% CI: −23.5 to −5.4) for boys, while no significant changes were observed in provinces (Figure 2c,d). Among fatal causes, traffic‐related fatalities increased significantly by 18.6% annually in girls in provinces (Figure S2B).

### Association between demographic/geographical factors and injuries during 2018–2022 in Mongolia

Children aged 1–4 years had significantly higher rates of non‐fatal injuries compared to infants aged 0–11 months, both in Ulaanbaatar (IRR = 2.01; 95% CI: 1.89–2.15) and provinces (IRR = 1.43; 95% CI: 1.32–1.56). Boys were more likely to sustain injuries than girls in Ulaanbaatar (IRR = 1.18; 95% CI: 1.12–1.24) and provinces (IRR = 1.32; 95% CI: 1.24–1.41). Injury rates were higher in all months except December, November and February compared to January. Injuries occurring at home were significantly more frequent than those outside the home in both Ulaanbaatar (IRR = 4.40; 95% CI: 4.18–4.63) and provinces (IRR = 3.71; 95% CI: 3.46–3.97) (Table 4).

**TABLE 4:** Association between demographic/geographical factors and injuries during 2018–2022 in Mongolia.

	Non‐fatal injury								Fatal injury							
	Ulaanbaatar				Province				Ulaanbaatar				Province			
	IRR	95% CI		*p* Value	IRR	95% CI		*p* Value	IRR	95% CI		*p* Value	IRR	95% CI		*p* Value
Age																
0–11 months	Reference															
1–4 years	2.01	1.89	2.15	0.000	1.43	1.32	1.56	0.000	0.94	0.67	1.32	0.720	0.79	0.66	0.94	0.009
Sex																
Female	Reference															
Male	1.18	1.12	1.24	0.000	1.32	1.24	1.41	0.000	0.96	0.71	1.31	0.810	1.02	0.85	0.86	1.206
Month																
January	Reference															
February	1.04	0.92	1.17	0.575	1.09	0.93	1.28	0.28	0.95	0.39	2.34	0.917	1.24	0.73	2.09	0.426
March	1.17	1.04	1.33	0.011	1.23	1.05	1.44	0.01	0.91	0.37	2.26	0.844	1.08	0.62	1.88	0.787
April	1.37	1.21	1.55	0.000	1.26	1.08	1.47	0.00	1.03	0.45	2.32	0.951	1.38	0.83	2.28	0.210
May	1.59	1.40	1.80	0.000	1.39	1.19	1.63	0.00	0.85	0.34	2.08	0.715	1.27	0.76	2.12	0.359
June	1.85	1.63	2.09	0.000	1.46	1.24	1.71	0.00	0.82	0.33	2.02	0.661	1.61	0.99	2.64	0.056
July	1.84	1.62	2.08	0.000	1.45	1.23	1.70	0.00	0.89	0.39	2.08	0.795	1.37	0.83	2.27	0.217
August	1.75	1.54	1.98	0.000	1.17	0.99	1.38	0.06	0.90	0.40	2.06	0.806	1.40	0.84	2.35	0.200
September	1.58	1.39	1.79	0.000	1.00	0.85	1.18	0.99	0.81	0.26	2.55	0.72	1.37	0.82	2.28	0.23
October	1.37	1.21	1.55	0.000	0.99	0.84	1.17	0.937	0.87	0.37	2.03	0.742	1.30	0.76	2.22	0.332
November	1.10	0.97	1.25	0.121	0.85	0.72	1.00	0.050	0.87	0.33	2.29	0.775	1.44	0.85	2.42	0.174
December	1.08	0.95	1.22	0.232	0.95	0.81	1.12	0.537	0.97	0.40	2.35	0.949	1.38	0.82	2.32	0.231
Location																
Outside	Reference															
Home	4.40	4.18	4.63	0.000	3.71	3.46	3.97	0.000	0.93	0.67	1.29	0.654	1.09	0.91	1.29	0.360

Abbreviations: CI, confidence interval; IRR, incidence rate ratio.

For fatal injuries, children aged 1–4 years in provinces (IRR = 0.79; 95% CI: 0.66–0.94) had a significantly lower mortality rate compared to those aged 0–11 months. Fatal injuries were equally distributed between boys and girls and showed no seasonal differences or significant variation by injury location (Table 4).

## DISCUSSION

Between 2018 and 2022, the incidence rate of non‐fatal injuries among Mongolian children under 5 years old in Ulaanbaatar showed an increasing trend, while the rate of fatal injuries decreased. In provinces, there was no significant change in the rates of non‐fatal or fatal injuries over time. Most non‐fatal injuries occurred at home, with higher incidence rates during summer, particularly in Ulaanbaatar, while fatal injury rates were consistently higher in rural provinces. Boys consistently exhibited higher injury incidence rates for both non‐fatal and fatal injuries. There were substantial provincial differences in injury rates.

The increasing rate of non‐fatal injuries among children under five in Ulaanbaatar can be attributed to a combination of socio‐economic challenges, infrastructure limitations and the pressures of urbanisation [22, 23, 27]. Urbanisation and rising living costs [31] have likely reduced parental supervision, as more parents work longer hours, contributing to higher injury incidence rates [32, 33]. This is reflected in the current study, where the majority of injuries occurred in domestic settings.

An important factor contributing to the high incidence of non‐fatal injuries in Ulaanbaatar could also be the accessibility of healthcare services and emergency care. Unlike rural provinces, Ulaanbaatar has well‐equipped hospitals and a specialised trauma centre, increasing the likelihood that non‐fatal injuries are recorded in the surveillance systems. Additionally, greater public awareness and proximity to medical facilities may encourage caregivers to seek treatment for minor injuries, leading to higher reporting rates. In contrast, rural regions with limited access to healthcare may experience significant underreporting of non‐fatal injuries, as caregivers may either manage minor injuries at home or face logistical challenges in seeking medical care in remote areas. This disparity in healthcare access and reporting may partly explain the observed higher non‐fatal injury rates in Ulaanbaatar compared to rural provinces. This suggests that future research should explore alternative data sources, such as household surveys, to gain a more comprehensive understanding of injury burdens in rural areas.

Conversely, the decline in fatal injuries in Ulaanbaatar may suggest that advances in medical technology, better emergency response systems and faster access to hospital care have increased survival rates for injured children [32]. Additionally, targeted public health interventions, such as road safety campaigns, child injury prevention programmes and stricter safety regulations in Ulaanbaatar, may have played a role in reducing fatality rates. However, while these interventions may have mitigated the severity of injuries, they have not necessarily reduced the overall burden of injuries, leading to an increase in non‐fatal cases.

Conversely, persistently high fatality rates in rural areas highlight challenges such as inadequate prehospital care, limited resources and long distances to health facilities, all of which hinder timely and effective treatment [33]. Provincial disparities in injury rates reveal the influence of local environmental and socioeconomic factors. For instance, provinces like Dornogovi and Govisumber, situated along the Trans‐Siberian Railway, and mining‐heavy regions like Umnugovi face heightened risks due to heavy transportation, industrial equipment and unsafe play areas. In sparsely populated provinces such as Zavkhan and Khentii, the lack of infrastructure and road access exacerbates the risk of injuries. Such regional variations emphasise the need for tailored interventions to address specific risk factors. Injuries are more common during the summer holidays in Mongolia, a period when older siblings often care for younger ones or children are left at home unsupervised due to kindergarten closures. Additionally, increased travel and outdoor activities during the warm season further elevate the risk of injuries during this time.

Falls, burns, injuries caused by inanimate and animate mechanical forces, and traffic‐related injuries were the top causes of non‐fatal injuries. Falls, particularly those caused by slipping or tripping, accounted for nearly half of all fall‐related injuries, consistent with global trends [36]. Young children's developing motor skills and lack of coordination make them particularly susceptible [37]. Burns, often caused by hot liquids or contact with cooking appliances, were the second leading cause of non‐fatal injuries. The finding was consistent with international settings that the physical abilities of children under five often exceed their cognitive understanding of the potential consequences of their actions [38]. In Mongolia, burns are more prevalent in ger areas, where the absence of a designated kitchen area exposes children to hazardous environments [11]. Injuries caused by being struck by inanimate mechanical forces were significant contributors to non‐fatal injuries.

Globally, the types and frequencies of injuries vary by region and country, influenced by environmental, socioeconomic, cultural and lifestyle factors [12, 37]. For example, rural areas often have higher exposure to farming equipment, heavy machinery and other hazards specific to agricultural settings [12], whereas urban areas are more likely to experience collisions with urban structures and furniture [39]. In Mongolia, rural areas report increased injuries linked to the use of imported machinery such as cement mixers and woodcutters, often operated without adequate safety measures [40, 41, 42]. Additionally, insufficient enforcement and inspection of safety standards in public and residential areas exacerbate risks for young children. Exposure to animate mechanical forces was the fifth leading cause of non‐fatal injuries. Injuries resulting from physical interactions, such as being hit, struck or scratched, are more common among children under five in Mongolia. Unlike cultures such as Japan, which prioritise patience and minimal physical interaction in public spaces, social interactions in Mongolia often involve more direct physical contact, potentially increasing the risk of such injuries [43]. In provinces, the prevalence of stray dogs, along with livestock such as horses, cows and camels, poses unique injury risks to children. These animals, often integral to rural livelihoods, create environments where children are more vulnerable to bites, kicks and other animal‐related injuries.

The findings reveal specific patterns in fatal injuries among children under five in Mongolia. Mortality due to suffocation, traffic injuries and drowning highlights critical safety concerns. Suffocation accounted for one‐third of all fatal injuries, with over half occurring in bed. This is likely linked to the common practice of bed‐sharing in Mongolia, where accidental suffocation may occur during breastfeeding. Traffic‐related fatalities were predominantly pedestrian‐related, often attributed to insufficient pedestrian signage, unclear zebra crossings, inadequate child supervision and drunk driving [44, 45]. Despite Mongolia's lack of sea access, drowning incidents were frequent, primarily resulting from children falling into rivers during unsupervised play. Caregivers in both rural areas and Ulaanbaatar often lock children inside homes while at work, which increases fire risks, as gers are typically heated with stoves or electric appliances. Unsupervised children may inadvertently start fires by playing with matches or other flammable materials [46, 47]. Falls from structures were a leading cause of death in Ulaanbaatar, reflecting inadequate fall‐prevention measures such as safety standards for balcony railings and window guards [48]. Additionally, bed‐related falls highlight a lack of bed rails, parental supervision and education on child safety.

### Limitation

The injury surveillance system in Mongolia provides crucial data on injuries among children under five; however, it has limitations in capturing more specific details about individual incidents. For example, 8531 cases (8.4% of the 101,731 total injury cases) in the dataset were classified as ‘undetermined intent,’ reflecting challenges in accurately identifying injury causes. Previous studies suggest misclassification of child fatalities, with some cases initially attributed to different illnesses later determined to result from injury or violence [2]. While this misclassification may slightly impact the precision of cause‐specific rates, it is unlikely to significantly alter the overall injury patterns observed in Mongolia. Furthermore, the lack of provincial live birth data during the study period prevented the calculation of fatal injury rates by province. As mentioned in the discussion, due to caregivers' health seeking behaviour and limited access to healthcare centres, non‐fatal injuries in rural areas may be underreported, potentially leading to an underestimation of injury rates compared to Ulaanbaatar. In addition, since the trauma surveillance system was introduced in 2017, it may have taken time to become fully implemented nationwide, particularly in rural areas. As a result, data from 2018 may still be affected by underreporting.

### Policy implications

To prevent injuries among children under five in Mongolia, targeted interventions addressing specific patterns and causes are essential. Most injuries could be prevented through focused strategies. Given that children are unable to control environmental risks, caregivers play a vital role in injury prevention [11]. However, Mongolia lacks a systemic approach to educating the public on injury prevention, particularly for the scattered population. This gap leaves many caregivers without crucial knowledge of injury prevention and first aid.

The findings highlight the importance of integrating safety education programmes into public health strategies, especially in high‐risk areas such as the ger areas in Ulaanbaatar and rural provinces that have limited healthcare infrastructure [4]. A comprehensive approach should prioritise parental education programmes to address key risks, such as unsafe nighttime breastfeeding practices like bed‐sharing with infants, and provide guidance on safe sleeping environments. Promoting safe cooking and heating practices in ger areas, and advising against leaving children locked at home unsupervised, are critical steps. Disseminating fire safety information widely is also crucial.

Fatal injuries disproportionately affect rural areas, underscoring the necessity of geographically and socioeconomically tailored interventions. Strengthening local health centre capacities and implementing community‐driven programmes can improve injury prevention and management in these regions. Moreover, the introduction of child death review mechanisms in some areas has started to provide a foundation for comprehensive prevention strategies, aiding policymakers in implementing effective measures [2]. Improving road safety requires enforcing stricter penalties for drunk driving, enhancing road signage with clear speed limits and pedestrian crossings, and creating child‐friendly pedestrian zones near schools and kindergarten. Emphasising the importance of adult supervision and life jacket use during water activities is also vital.

Public health campaigns should account for seasonal injury trends, particularly the increased risk during summer months. Building safety regulations must also be enforced to ensure compliance with standards for window guards and staircase protections in new constructions. Finally, tailored awareness campaigns for ger area residents can help promote the adoption of safety measures suited to their unique living environments.

### Conclusion

Non‐fatal injury rates among children under five in Mongolia are increasing, particularly in Ulaanbaatar, even as fatal injury rates show a declining trend. Injury rates vary significantly across rural provinces, with higher rates in certain areas driven by specific socioeconomic factors. Fatal injury rates remain disproportionately higher in rural regions, highlighting the need for targeted safety interventions that address the unique risks in rural settings. Tailored strategies are essential to mitigate specific causes of injuries in Ulaanbaatar and the provinces.

## CONFLICT OF INTEREST STATEMENT

The authors declare no conflicts of interest.

## Supporting information


**Data S1.** Supporting Information.

## References

[1] Institute for Health Metrics and Evaluation . Global burden of diseases results [Internet]. 2019 [cited 2023 Dec 25]. Available from: https://vizhub.healthdata.org/gbd-results

[2] The World Health Organization, Western Pacific Region . Situation analysis of well‐child care in Mongolia [Internet]. Ulaanbaatar, Mongolia; 2022. [cited 2024 Feb 13]. Available from: https://iris.who.int/bitstream/handle/10665/375903/9789290620181-eng.pdf?sequence=1

[3] The World Health Organization and Health Development Center Mongolia . Health indicators Mongolia, 2023 [Internet]. Health Development Center Mongolia [cited 2024 Nov 19]. 2023. Available from: https://hdc.gov.mn/media/files/2023%20uzuulelt.pdf

[4] Xu Y , Zhang Y , Chen J . Migration under economic transition and changing climate in Mongolia. J Arid Environ. 2021;185:104333.

[5] Macrotrends . Mongolia unemployment rate 1991–2024 [Internet]. [cited 2024 Jan 22]. Available from: https://www.macrotrends.net/countries/MNG/mongolia/unemployment-rate

[6] Caldieron JM . Ger districts in Ulaanbaatar, Mongolia: housing and living condition surveys. Int J Innov Appl Stud. 2013;4:465–476.

[7] The World Bank and National Statistics Office of Mongolia . World Bank. Mongolia's 2020 poverty rate estimated at 27.8 percent. 2021. [cited 2024 Jan 11]. Available from: https://www.worldbank.org/en/news/press‐release/2021/12/30/mongolia‐s‐2020‐poverty‐rate‐estimated‐at‐27‐8‐percent

[8] Data Commons . Mongolia – place explorer – data commons [Internet]. [cited 2024 Jan 22]. Available from: https://datacommons.org/place/country/MNG/?utm_medium=explore&mprop=amount&popt=EconomicActivity&cpv=activitySource,GrossDomesticProduction&hl=en

[9] Altangerel P , Damdinbazar O , Borgilchuluun U , Batbayar D , Erdenebat B , Purevdorj T , et al. An exploration of productivity costs and years of potential life lost: understanding the impact of premature mortality from injury in Mongolia. Health Serv Insights. 2023;16:11786329231212295. 10.1177/11786329231212295 38028123 PMC10666683

[10] Pynn ER , Tsogzolbaatar EO , Davison CM . Childhood burn injury in the ger districts of Ulaanbaatar, Mongolia: an analysis of parent narratives. Int J Injury Control Saf Promot. 2024;31(4):1–10. 10.1080/17457300.2024.2392266 39166833

[11] Gerelmaa G , Tumen‐Ulzii B , Nakahara S , Ichikawa M . Patterns of burns and scalds in Mongolian children: a hospital‐based prospective study. Trop Med Int Health. 2018;23(3):334–340. 29352506 10.1111/tmi.13034

[12] Dhage VD , Nagtode N . Health problems among under‐five age group children in developing countries: a narrative review. Cureus. 2024;16(2):e55019. 38550476 10.7759/cureus.55019 PMC10973914

[13] State Great Hural of Mongolia . Mongolia sustainable development vision 2030. 2016. [cited 2024 Mar 10]. Available from: https://faolex.fao.org/docs/pdf/mon184386.pdf

[14] World Population Review . Mongolia population 2024. 2024. [cited 2024 Jan 11]. Available from: https://worldpopulationreview.com/countries/mongolia-population

[15] YНДЭСНИЙ СТАТИСТИКИЙН ХОРОО . МОНГОЛ УЛСАД ОРШИН СУУГАА ХYН АМЫН ТОО насны бүлэг, баг/хороогоор [Internet]. [cited 2022 Feb 13]. Available from: https://www.1212.mn/tables.aspx?tbl_id=DT_NSO_0300_067V2&BAG_select_all=0&BAGSingleSelect=_51122_51119_51116_51113_51110_51101_51104&AGE_GROUP5_select_all=0&AGE_GROUP5SingleSelect=_2&YearH_select_all=0&YearHSingleSelect=_202102&YearY_select_all=0&YearYSingleSelect=&viewtype=table

[16] The Government of Mongolia . Legal portal. Health care and service act of Mongolia. 2016. [cited 2025 Mar 10]. Available from: https://legalinfo.mn/

[17] Ministry of Health Mongolia (MoH) . Regional Diagnostic Centers in Mongolia. [cited 2024 Nov 19]. Available from: https://www.moh.gov.mn:443/p/69

[18] Mongolian Parliament . There are 58 sums with a population of less than 2000 and 54 communes with a population of more than 10,000. Mongolian Parliament News. [cited 2022 Dec 19]. 2022. Available from: https://www.uih.mn/n/175

[19] Holtz J , Associates A , Noh KM . Regulation of private primary health care in Mongolia a country assessment report. 2018. [cited 2023 Apr 20]. Available from: https://www.jointlearningnetwork.org/wp-content/uploads/2021/04/Mongolia_PSE_Assessment_R1.pdf

[20] Oikon . 100 of Mongolia's 330 soums are connected to the provincial capital by paved roads [Internet]. 2021 [cited 2022 Dec 19]. Available from: https://ikon.mn/n/2e32

[21] Jigjidsuren A , Byambaa T , Altangerel E , Batbaatar S , Saw YM , Kariya T , et al. Free and universal access to primary healthcare in Mongolia: the service availability and readiness assessment. BMC Health Serv Res. 2019;19(1):129. 10.1186/s12913-019-3932-5 30786897 PMC6381625

[22] Ministry of Health Mongolia . A/814 order of Minister of Health. 2021. [cited 2025 Mar 10]. Available from: https://www.hdc.gov.mn/media/uploads/2022-01/A814.pdf

[23] National Statistics Office of Mongolia . Population of Mongolia; 2023. [cited 2023 Dec 28]. Available from: https://1212.mn/en

[24] Health development center and World Health Organization Western Pacific region . Health indicators Mongolia, 2019.pdf [Internet]. 2019. [cited 2024 Mar 10]. Available from: http://hdc.gov.mn/media/uploads/2020‐08/2019‐eruul_mendin_uzuulelt_MU_mail.indd_2020_______7___21final.pdf

[25] Health Development Center , World Health Organization Western Pacific Region . Health indicator, Mongolia, 2018 [Internet]. Ulaanbaatar, Mongolia. 2019. [cited 2019 Nov 7]. Available from: http://www.hdc.gov.mn/media/files/2018_t5E7aGV.pdf

[26] Health Development Center, World Health Organization . Health indicators, Mongolia, 2021 [Internet]. 2021 [cited 2022 Nov 1]. Available from: http://hdc.gov.mn/media/uploads/2022-05/ERUUL_MENDIIN_UZUULELT_2021.pdf

[27] Health Development Center and World Health Organization Western Pacific Region . Health indicators Mongolia. 2022. 2022. [cited 2023 Dec 28]. Available from: http://hdc.gov.mn/media/files/2022.pdf

[28] Health Development Center and World Health Organization Western Pacific Region . Health indicators Mongolia, 2020.pdf; 2020. [cited 2023 Dec 28]. Available from: http://hdc.gov.mn/media/files/2020_WKG0S7t.pdf

[29] National Cancer Institute of USA . Joinpoint trend analysis software. Joinpoint regression program. 2023. [cited 2023 Dec 28]. Available from: https://surveillance.cancer.gov/joinpoint/

[30] Kim HJ , Fay MP , Feuer EJ , Midthune DN . Permutation tests for joinpoint regression with applications to cancer rates. Stat Med. 2000;19(3):335–351. 10649300 10.1002/(sici)1097-0258(20000215)19:3<335::aid-sim336>3.0.co;2-z

[31] The Asian Development Bank . Mongolia: urban sector fact sheet. 2022. [cited 2024 Nov 11]. Available from: https://www.adb.org/publications/mongolia-urban-sector-fact-sheet

[32] Asian Development Bank . Ulaanbaatar urban services and ger areas development investment program – Tranche 2. 2022 Jan. Report No.: October–December 2021 [Internet]. Available from: https://www.adb.org/sites/default/files/project-documents/45007/45007-005-eapr-en_9.pdf

[33] World Bank . Urban poverty in Ulaanbaatar final. 2017. [cited 2024 Jan 11]. Available from: https://thedocs.worldbank.org/en/doc/459481506972842865‐0070022017/original/UrbanPovertyinUlaanbaatarFinal20170810.pdf

[34] The Asian Development Bank. Mongolia . Improving access to health services for disadvantaged groups investment program [Internet]. 2019 [cited 2024 Nov 11]. Available from: https://www.adb.org/projects/49173-003/main

[35] Lombardo S , Unurbileg B , Gerelmaa J , Bayarbaatar L , Sarnai E , Price R . Trauma care in Mongolia: INTACT evaluation and recommendations for improvement. World J Surg. 2018;42(8):2285–2292. 29387959 10.1007/s00268-018-4462-8

[36] He S , Lunnen JC , Puvanachandra P , Amar‐Singh , Zia N , Hyder AA . Global childhood unintentional injury study: multisite surveillance data. Am J Public Health. 2014;104(3):e79–e84. 24432924 10.2105/AJPH.2013.301607 PMC3953760

[37] Jacobs JV . A review of stairway falls and stair negotiation: lessons learned and future needs to reduce injury. Gait Posture. 2016;49:159–167. 27427833 10.1016/j.gaitpost.2016.06.030

[38] Sanders JE , Mogilner L . Child safety and injury prevention. Pediatrics. 2015;36(6):268–269. 10.1542/pir.36-6-268 26034259

[39] Yao M , Wu G , Zhao Z , Luo M , Zhang J . Unintentional injury mortality among children under age five in urban and rural areas in the Sichuan province of west China, 2009–2017. Sci Rep. 2019;9(1):2963. 30814522 10.1038/s41598-019-38936-6 PMC6393442

[40] MONTSAME News Agency . An exhibition specializing in agricultural and road construction machinery and equipment has opened [Internet]. 2021 [cited 2024 Nov 11]. Available from: https://montsame.mn/mn/read/276258

[41] National Center for Road Transport . Study on the feasibility and economic efficiency of manufacturing and assembling vehicles in Mongolia 2020–2021. 2021. [cited 2024 Nov 11]. Available from: https://www.transdep.mn/content_img/2022/Mongol_ulsad_mashin_uildverlekh_ugsrakh_b.pdf

[42] The World Bank . Mongolia economic monitor [Internet]. 2018 [cited 2024 Nov 11]. Available from: https://thedocs.worldbank.org/en/doc/537691530843746159‐0070022018/original/MongoliaEconomicMonitorMongolianlanguage.pdf

[43] Culture crossing . Personal space & touching. [cited 2024 Nov 19]. Available from: https://guide.culturecrossing.net/basics_business_student_details.php?Id=9&CID=138

[44] Gantugs I . In the past six years, 371 children and 1991 adults have died in road traffic accidents. 2024. [cited 2024 Nov 19]. Available from: https://itoim.mn/a/2024/04/18/society/pcg

[45] Police Department Mongolia . Let's prevent young children from being involved in road traffic accidents and incidents [Internet]. 2022 [cited 2024 Nov 19]. Available from: https://police.gov.mn/a/5925?fbclid=IwAR1nMM8ZVT_LriBNLVQqNhyCXIa3pg1y9DYgXPb9ytj‐OiuqMUVMX0jYgbU

[46] Ikon . Let's avoid locking children inside the home. [cited 2024 Nov 19]. Available from: https://ikon.mn/n/h4s

[47] Шуурхай.мн . Four children aged 2 to 7 died in a ger fire. 2020. [cited 2024 Nov 19]. Available from: https://shuurhai.mn/p/12028

[48] Naranchuluun G . Within three days, three young children have died from falling from heights. 2023. [cited 2024 Nov 19]. Available from: https://mongoltv.mn/post/132967

