# Supplementary material for: Trends and patterns of injuries among children under five in Mongolia: A retrospective analysis of national injury surveillance data between 2018 and 2022
Source: Trop Med Int Health. 2025 May 8;30(7):640–51. doi: 10.1111/tmi.14117 (PMC12213320; doi:10.1111/tmi.14117)
Supplement: Supplementary file 1 — Data S1. Supporting Information. [file TMI-30-640-s001.pdf]

Supplementary Table 1. Reported causes of non-fatal and fatal injuries by geographic area

Causes of injury	Ulaanbaatar			Provinces			Total		
	N	%	IR	N	%	IR	N	%	IR
**Non-fatal injury**									
Falls	41,721	46.79%	469.14	4,501	38.01%	44.52	46,222	45.76%	243.23
Fire or hot object or substance	12,636	14.17%	142.09	4,064	34.32%	40.20	16,700	16.53%	87.88
Struck by or against inanimate mechanical force	12,241	13.73%	137.65	1,458	12.31%	14.42	13,699	13.56%	72.09
Exposure to animate mechanical forces	11,583	12.99%	130.25	556	4.70%	5.50	12,139	12.02%	63.88
Undetermined intent	6,931	7.77%	77.94		0.00%	-	6,931	6.86%	36.47
Traffic injury	2,956	3.31%	33.24	758	6.40%	7.50	3,714	3.68%	19.54
Poisoning	346	0.39%	3.89	196	1.66%	1.94	542	0.54%	2.85
Suffocation	210	0.24%	2.36	79	0.67%	0.78	289	0.29%	1.52
Assault	202	0.23%	2.27	49	0.41%	0.48	251	0.25%	1.32
Exposure to forces of nature	93	0.10%	1.05	85	0.72%	0.84	178	0.18%	0.94
Exposure to electric current, radiation and extreme ambient air temperature and pressure	139	0.16%	1.56	8	0.07%	0.08	147	0.15%	0.77
Contact with venomous animals and plants	36	0.04%	0.40	64	0.54%	0.63	100	0.10%	0.53
Overexertion	65	0.07%	0.73		0.00%	-	65	0.06%	0.34
Drawning	11	0.01%	0.12	20	0.17%	0.20	31	0.03%	0.16
Complications of medical and surgical care	5	0.01%	0.06	1	0.01%	0.01	6	0.01%	0.03
Exposure to other specified factors		0.00%	-	1	0.01%	0.01	1	0.00%	0.01
Exposure to unspecified factor		0.00%	-	1	0.01%	0.01	1	0.00%	0.01
**Total**	**89,175**	**100.0%**	1,002.75	**11,841**	**100.0%**	117.11	**101,016**	**100.0%**	531.56
**Fatal injury**									
Suffocation	54	31.95%	0.28	194	35.53%	1.10	248	34.69%	0.67
Traffic injury	26	15.38%	0.13	122	22.34%	0.69	148	20.70%	0.40
Drawning	8	4.73%	0.04	90	16.48%	0.51	98	13.71%	0.27
Fire or hot object or substance	16	9.47%	0.08	50	9.16%	0.28	66	9.23%	0.18
Falls	18	10.65%	0.09	24	4.40%	0.14	42	5.87%	0.11
Poisoning	31	18.34%	0.16	11	2.01%	0.06	42	5.87%	0.11
Struck by or against inanimate mechanical force	3	1.78%	0.02	16	2.93%	0.09	19	2.66%	0.05
Assault	1	0.59%	0.01	12	2.20%	0.07	13	1.82%	0.04
Exposure to forces of nature	2	1.18%	0.01	10	1.83%	0.06	12	1.68%	0.03
Undetermined intent	5	2.96%	0.03	2	0.37%	0.01	7	0.98%	0.02
Contact with venomous animals and plants	1	0.59%	0.01	5	0.92%	0.03	6	0.84%	0.02
Exposure to electric current, radiation and extreme ambient air temperature and pressure	3	1.78%	0.02	2	0.37%	0.01	5	0.70%	0.01
Complications of medical and surgical care	1	0.59%	0.01	3	0.55%	0.02	4	0.56%	0.01
Exposure to animate mechanical forces		0.00%	-	3	0.55%	0.02	3	0.42%	0.01
Exposure to unspecified factor		0.00%	-	1	0.18%	0.01	1	0.14%	0.00
Lifestyle-related condition		0.00%	-	1	0.18%	0.01	1	0.14%	0.00
**Total**	**169**	**100.00%**	**0.87**	**546**	**100.00%**	**3.10**	**715**	**100.00%**	**1.94**

N- Number of injury cases

% - Percentage of injuries among all-cause non-fatal injuries

IR- Incidence Rate (per 10,000 U5 children)

Supplementary Table 2. Detail causes of five leading causes of non-fatal injuries during 2018-2022 in Mongolia

Causes of	Ulaanbaatar		Provinces		Total	
**FALL**	N	%	N	%	N	%
Fall on same level from slipping, tripping and stumbling	19,745	47.33%	1,905	42.32%	21,650	46.84%
Fall from bed	9,352	22.42%	1,028	22.84%	10,380	22.46%
Fall from chair	2,239	5.37%	380	8.44%	2,619	5.67%
Fall on and from playground equipment	1,939	4.65%	149	3.31%	2,088	4.52%
Fall from other furniture	1,804	4.32%	126	2.80%	1,930	4.18%
Other fall from one level to another	1,773	4.25%	206	4.58%	1,979	4.28%
Fall on and from stairs and steps	1,570	3.76%	194	4.31%	1,764	3.82%
Other fall on same level due to collision with another person	760	1.82%	153	3.40%	913	1.98%
Fall while being carried or supported by other persons	651	1.56%	56	1.24%	707	1.53%
Fall involving ice-skates, skis, roller-skates or skateboards	501	1.20%	17	0.38%	518	1.12%
Other slipping, tripping and stumbling and falls	454	1.09%	134	2.98%	588	1.27%
Unspecified fall	412	0.99%	37	0.82%	449	0.97%
Fall from, out of or through building or structure	306	0.73%	31	0.69%	337	0.73%
Fall due to ice and snow	145	0.35%	31	0.69%	176	0.38%
Fall from non-moving wheelchair, nonmotorized scooter and motorized mobility scooter	25	0.06%	3	0.07%	28	0.06%
Fall from tree	17	0.04%	11	0.24%	28	0.06%
Fall from cliff	16	0.04%	23	0.51%	39	0.08%
Fall on and from ladder	7	0.02%	9	0.20%	16	0.03%
Fall, jump or diving into water	4	0.01%	1	0.02%	5	0.01%
Fall on and from scaffolding	1	0.00%	6	0.13%	7	0.02%
Falling, jumping or pushed from a high place, undetermined intent		0.00%	1	0.02%	1	0.00%
**Total**	**41,721**	**100%**	**4,501**	**100%**	**46,222**	**100%**
**FIRE OR HOT OBJECT OR SUBSTANCE**						
Contact with hot drinks, food, fats and cooking oils	5,425	42.93%	2,398	59.01%	7,823	46.84%
Contact with hot tap-water	3,417	27.04%	728	17.91%	4,145	24.82%
Contact with hot household appliances	1,907	15.09%	511	12.57%	2,418	14.48%
Contact with hot heating appliances, radiators and pipes	1,035	8.19%	74	1.82%	1,109	6.64%
Contact with steam and other hot vapors	285	2.26%	37	0.91%	322	1.93%
Exposure to other specified smoke, fire and flames	276	2.18%	25	0.62%	301	1.80%
Contact with other heat and hot substances	123	0.97%	27	0.66%	150	0.90%
Exposure to uncontrolled fire in building or structure	55	0.44%	3	0.07%	58	0.35%
Exposure to unspecified smoke, fire and flames	35	0.28%	8	0.20%	43	0.26%
Contact with other hot fluids	29	0.23%	202	4.97%	231	1.38%
Contact with hot engines, machinery and tools	24	0.19%	20	0.49%	44	0.26%
Exposure to ignition of highly flammable material	12	0.09%	14	0.34%	26	0.16%
Contact with hot air and other hot gases	6	0.05%	4	0.10%	10	0.06%
Contact with other hot metals	5	0.04%	11	0.27%	16	0.10%
Exposure to controlled fire in building or structure	1	0.01%		0.00%	1	0.01%
Exposure to controlled fire, not in building or structure	1	0.01%		0.00%	1	0.01%
Exposure to uncontrolled fire, not in building or structure		0.00%	1	0.02%	1	0.01%
Exposure to ignition or melting of nightwear		0.00%	1	0.02%	1	0.01%
**Total**	**12,636**	**100.00%**	**4,064**	**100.00%**	**16,700**	**100.00%**
**EXPOSURE TO ANIMATE MECHANICAL FORCE**						
Accidental hit, strike, kick, twist, bite or scratch by another person	9,989	86.24%	46	8.27%	10,035	82.67%
Contact with dog	1,245	10.75%	278	50.00%	1,523	12.55%
Contact with other mammals	234	2.02%	124	22.30%	358	2.95%
Accidental striking against or bumped into by another person	41	0.35%	48	8.63%	89	0.73%
Contact with other nonvenomous reptiles	44	0.38%	29	5.22%	73	0.60%
Bitten or stung by nonvenomous insect and other nonvenomous arthropods	23	0.20%	20	3.60%	43	0.35%
Contact with nonvenomous plant thorns and spines and sharp leaves	6	0.05%	1	0.18%	7	0.06%
Crushed, pushed or stepped on by crowd or human stampede	1	0.01%	5	0.90%	6	0.05%
Exposure to other animate mechanical forces		0.00%	4	0.72%	4	0.03%
Contact with rodent		0.00%	1	0.18%	1	0.01%
**Total**	**11,583**	**100.00%**	**556**	**100.00%**	**12,139**	**100.00%**
**STRUCK OR BY AGAINST UNANIMATE MECHANICAL FORCE**						
Striking against or struck by other objects	6,082	49.69%	311	21.33%	6,393	46.67%
Caught, crushed, jammed or pinched in or between objects	2,124	17.35%	247	16.94%	2,371	17.31%
Struck by thrown, projected or falling object	1,305	10.66%	152	10.43%	1,457	10.64%
Contact with lifting and transmission devices, not elsewhere classified	934	7.63%	7	0.48%	941	6.87%
Contact with sharp glass	766	6.26%	154	10.56%	920	6.72%
Foreign body entering into or through a natural orifice	309	2.52%	368	25.24%	677	4.94%
Contact with other sharp objects	275	2.25%	77	5.28%	352	2.57%
Contact with nonpowered hand tool	158	1.29%	52	3.57%	210	1.53%
Striking against or struck by sports equipment	97	0.79%	10	0.69%	107	0.78%
Contact with other powered hand tools and household machinery	87	0.71%	20	1.37%	107	0.78%
Foreign body or object entering through skin	79	0.65%	16	1.10%	95	0.69%
Exposure to other inanimate mechanical forces	13	0.11%	28	1.92%	41	0.30%
Contact with other and unspecified machinery		0.00%	7	0.48%	7	0.05%
Contact with powered lawn mower	3	0.02%	3	0.21%	6	0.04%

Contact with agricultural machinery		0.00%	3	0.21%	3	0.02%
Explosion and rupture of pressurized tire, pipe or hose	2	0.02%	1	0.07%	3	0.02%
Explosion of other materials	2	0.02%	1	0.07%	3	0.02%
Accidental discharge and malfunction from other and unspecified firearms and guns	2	0.02%		0.00%	2	0.01%
Explosion and rupture of other specified pressurized devices	2	0.02%		0.00%	2	0.01%
Exposure to vibration	1	0.01%		0.00%	1	0.01%
Contact with hypodermic needle		0.00%	1	0.07%	1	0.01%
**Total**	**12,241**	**100.00%**	**1,458**	**100.00%**	**13,699**	**100.00%**

TRAFFIC INJURY

Car occupant injured in transport accident	1,817	61.47%	366	48.28%	2,183	58.78%
Pedestrian injured in transport accident	772	26.12%	141	18.60%	913	24.58%
Other land transport accidents	194	6.56%	67	8.84%	261	7.03%
Motorcycle rider injured in transport accident	71	2.40%	118	15.57%	189	5.09%
Pedal cycle rider injured in transport accident	37	1.25%	48	6.33%	85	2.29%
Occupant of pick-up truck or van injured in transport accident	24	0.81%	9	1.19%	33	0.89%
Bus occupant injured in transport accident	26	0.88%	2	0.26%	28	0.75%
Occupant of heavy transport vehicle injured in transport accident	11	0.37%	6	0.79%	17	0.46%
Accidental non-transport drowning and submersion	4	0.14%		0.00%	4	0.11%
Occupant of three-wheeled motor vehicle injured in transport accident		0.00%	1	0.13%	1	0.03%
**Total**	**2,956**	**100.00%**	**758**	**100.00%**	**3,714**	**100.00%**

Supplementary Figure 1. Trend of incidence rate and average annual percentage change of leading 5 causes of non-fatal injuries

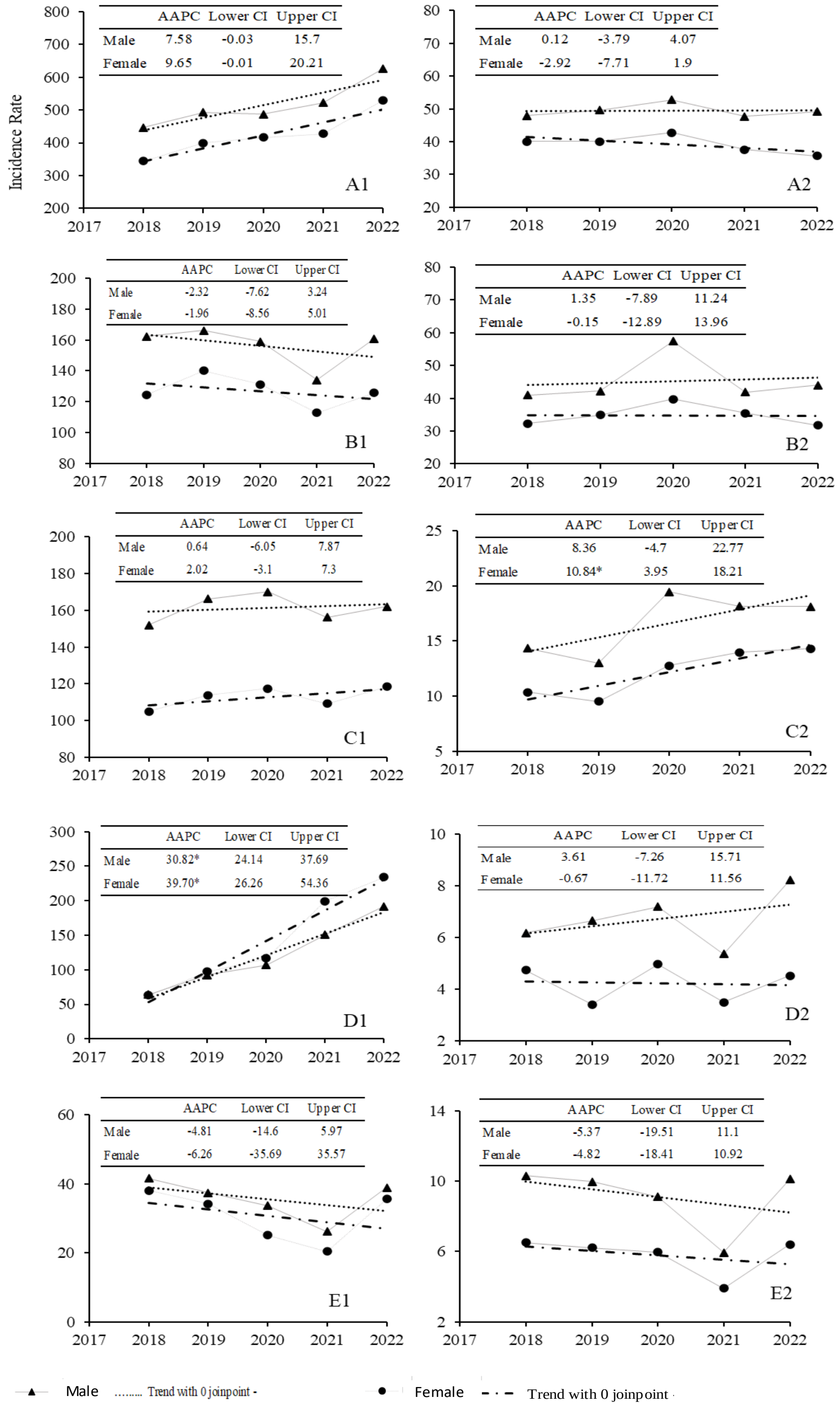

1- Ulaanbaatar; 2 Provinces
A- Fall, B- Fire and hot substances, C- Struck, D- Exposure to animate mechanical force; E- Traffic injury

AAPC -Average Annual Percentage Changes of injuries bewteen 2018 and 2022
* Signifcant AAPC
Lower CI - Lower Confidence Interval
Upper CI - Upper Confidence Interval

Supplementary Table 3. Detail causes of five leading causes of fatal injuries during 2018-2022 in Mongolia

Causes of	Ulaanbaatar		Province		Total	
**SUFFOCATION**	N	%	N	%	N	%
Accidental suffocation and strangulation in bed	27	50.00%	102	52.58%	129	52.02%
Inhalation and ingestion of food causing obstruction of respirator	15	27.78%	35	18.04%	50	20.16%
Inhalation of gastric contents	2	3.70%	22	11.34%	24	9.68%
Inhalation and ingestion of other objects causing obstruction of r	7	12.96%	14	7.22%	21	8.47%
Unspecified threat to breathing	1	1.85%	7	3.61%	8	3.23%
Other accidental hanging and strangulation	1	1.85%	6	3.09%	7	2.82%
Other specified threats to breathing	1	1.85%	4	2.06%	5	2.02%
Confined to or trapped in a low-oxygen environment		0.00%	3	1.55%	3	1.21%
Threat to breathing due to cave-in, falling earth and other substances		0.00%	1	0.52%	1	0.40%
**Total**	**54**	**100%**	**194**	**100%**	**248**	**100%**
**TRAFFIC INJURY**						
Pedestrian injured in transport accident	23	88.46%	102	83.61%	125	84.46%
Car occupant injured in transport accident	2	7.69%	13	10.66%	15	10.14%
Motorcycle rider injured in transport accident		0.00%	5	4.10%	5	3.38%
Occupant of pick-up truck or van injured in transport accident	1	3.85%		0.00%	1	0.68%
Occupant of heavy transport vehicle injured in transport accident		0.00%	1	0.82%	1	0.68%
Traffic accident of specified type but victim's mode of transport unknown		0.00%	1	0.82%	1	0.68%
**Total**	**26**	**100.00%**	**122**	**100.00%**	**148**	**100.00%**
**DRAWNING**						
Drowning and submersion following fall into natural water	6	75.00%	31	34.44%	37	37.76%
Unspecified drowning and submersion	1	12.50%	28	31.11%	29	29.59%
Drowning and submersion while in natural water		0.00%	23	25.56%	23	23.47%
Other specified drowning and submersion		0.00%	4	4.44%	4	4.08%
Drowning and submersion while in bath-tub		0.00%	1	1.11%	1	1.02%
Drowning and submersion following fall into bath-tub	1	12.50%		0.00%	1	1.02%
Drowning and submersion while in swimming-pool		0.00%	1	1.11%	1	1.02%
Drowning and submersion following fall into swimming-pool		0.00%	1	1.11%	1	1.02%
Drowning and submersion, undetermined intent		0.00%	1	1.11%	1	1.02%
**Total**	**8**	**100.00%**	**90**	**100.00%**	**98**	**100.00%**
**FIRE OR HOT OBJECT OR SUBSTANCE**						
Exposure to other specified smoke, fire and flames	2	12.50%	18	36.00%	20	30.30%
Contact with hot drinks, food, fats and cooking oils	11	68.75%	8	16.00%	19	28.79%
Exposure to uncontrolled fire in building or structure		0.00%	14	28.00%	14	21.21%
Contact with hot tap-water	3	18.75%	3	6.00%	6	9.09%
Exposure to ignition of highly flammable material		0.00%	3	6.00%	3	4.55%
Exposure to controlled fire in building or structure		0.00%	2	4.00%	2	3.03%
Exposure to unspecified smoke, fire and flames		0.00%	2	4.00%	2	3.03%
**Total**	**16**	**100.00%**	**50**	**100.00%**	**66**	**100.00%**
**FALL**						
Fall from, out of or through building or structure	13	72.22%	5	20.83%	18	42.86%
Fall from bed	1	5.56%	5	20.83%	6	14.29%
Other slipping, tripping and stumbling and falls	2	11.11%	4	16.67%	6	14.29%
Fall on same level from slipping, tripping and stumbling	1	5.56%	3	12.50%	4	9.52%
Other fall from one level to another		0.00%	3	12.50%	3	7.14%
Unspecified fall		0.00%	2	8.33%	2	4.76%
Fall from other furniture	1	5.56%		0.00%	1	2.38%
Fall while being carried or supported by other persons		0.00%	1	4.17%	1	2.38%
Fall from cliff		0.00%	1	4.17%	1	2.38%
**Total**	**18**	**100.00%**	**24**	**100.00%**	**42**	**100.00%**
**POISONING**						
Accidental poisoning by and exposure to other gases	22	70.97%	2	18.18%	24	57.14%
Accidental poisoning by and exposure to other and unspecified drugs, medicaments and biological substances	4	12.90%	4	36.36%	8	19.05%

Accidental poisoning by and exposure to other and unspecified chemicals and noxious substances	2	6.45%	1	9.09%	3	7.14%
Accidental poisoning by and exposure to nonopioid analgesics, antipyretics and antirheumatics	1	3.23%	1	9.09%	2	4.76%
Accidental poisoning by and exposure to pesticides	1	3.23%	1	9.09%	2	4.76%
Accidental poisoning by and exposure to alcohol	1	3.23%			1	2.38%
Accidental poisoning by and exposure to organic solvents and halogenated hydrocarbons and their vapours			1	9.09%	1	2.38%
Poisoning by and exposure to other and unspecified chemicals and noxious substances, undetermined intent		0.00%	1	9.09%	1	2.38%
**Total**	**31**	**100.00%**	**11**	**100.00%**	**42**	**100.00%**

Supplementray Figure 2. Trend of average annual percentage change of two leading causes of fatal injuries

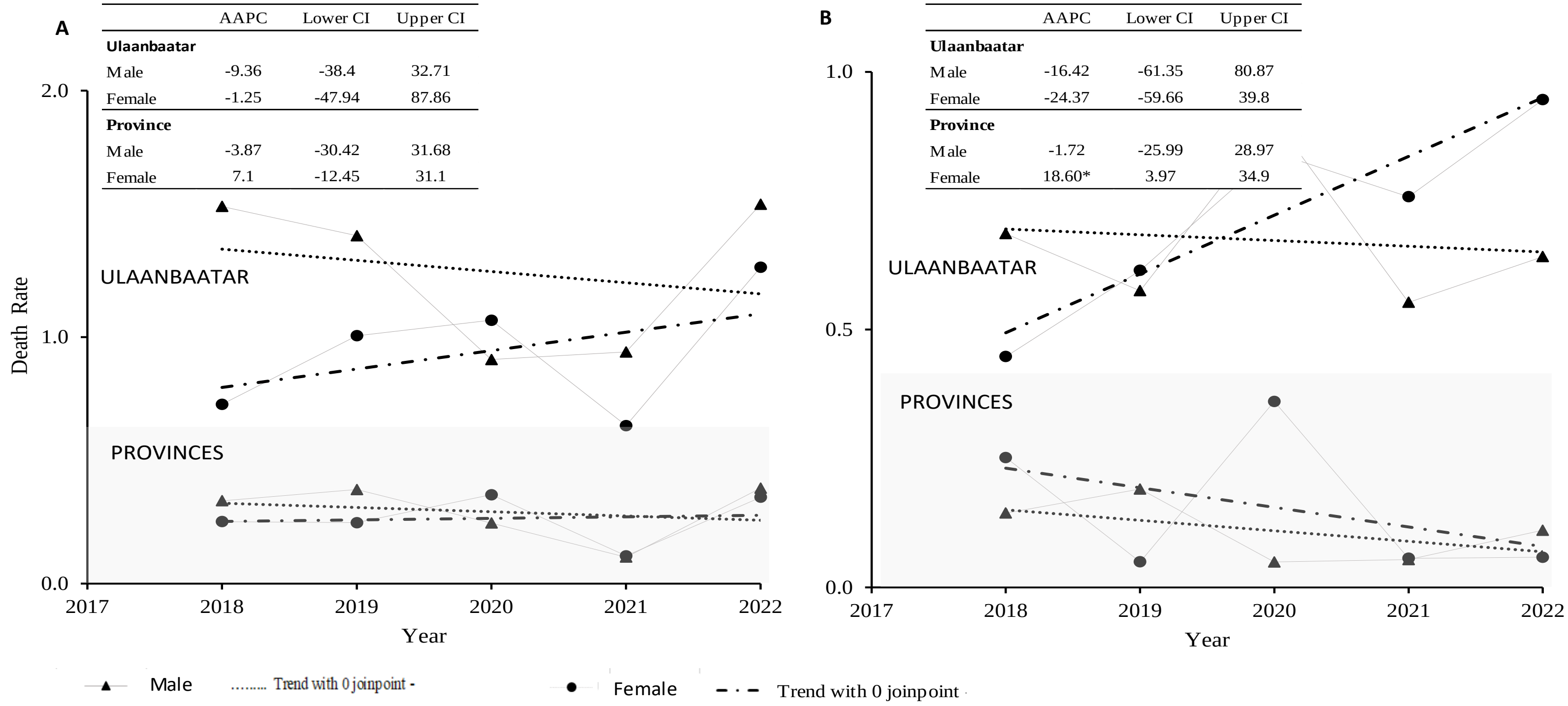

A

	AAPC	Lower CI	Upper CI
**Ulaanbaatar**			
Male	-9.36	-38.4	32.71
Female	-1.25	-47.94	87.86
**Province**			
Male	-3.87	-30.42	31.68
Female	7.1	-12.45	31.1

B

	AAPC	Lower CI	Upper CI
**Ulaanbaatar**			
Male	-16.42	-61.35	80.87
Female	-24.37	-59.66	39.8
**Province**			
Male	-1.72	-25.99	28.97
Female	18.60*	3.97	34.9

A- Suffocation, B- Traffic Injury

AAPC -Average Annual Percentage Changes of injuries bewteen 2018 and 2022
* Signifcant AAPC
Lower CI - Lower Confidence Interval
Upper CI - Upper Confidence Interval
Only top two causes were analyzed due to few number of fatal injuries for other specific causes
